# Supplementary material for: Antimicrobial Activity of Pinus wallachiana Leaf Extracts against Fusarium oxysporum f. sp. cubense and Analysis of Its Fractions by HPLC
Source: Pathogens. 2022 Mar 12;11(3):347. doi: 10.3390/pathogens11030347 (PMC8953374; doi:10.3390/pathogens11030347)
Supplement: Supplementary file 1 [file pathogens-11-00347-s001.zip › pathogens-1605241-supplementary.pdf]

Supplementary Table S1: Completely randomized analysis of variance for percent inhibition of Foc mycelial growth using four fractions of *P. wallachiana*, prepared from liquid-liquid fractionation. (p< 0.05)

Source	DF	SS	MS	F	P
Treatment	7	61937.7	8848.24	29886	0.0000
Error	32	9.5	0.30		
Total	39	61947.2			

Grand Mean 37.768 CV 1.44

Supplementary Table S2: Pairwise Comparisons Test of Percent Inhibition for four fractions of *P. wallachiana* against Foc

Treatment	Mean	Homogeneous Groups
8	100.00	A
4	75.956	B
2	68.926	C
6	57.258	D
1	0.0000	E
3	0.0000	E
5	0.0000	E
7	0.0000	E

Alpha 0.05 Standard Error for Comparison 0.3441
Critical T Value 2.037 Critical Value for Comparison 0.7010
There are 5 groups (A, B, etc.) in which the means
are not significantly different from one another.

Supplementary Table S3: Completely randomized analysis of variance for zone of inhibition produced by four different fractions of *P. wallachiana* against Foc. (p< 0.05)

Source	DF	SS	MS	F	P
Treatment	7	4925.78	703.682	440	0.0000
Error	32	51.20	1.600		
Total	39	4976.97			

Grand Mean 10.975 CV 11.53

Supplementary Table S4: Pairwise Comparisons Test of ZOI for four fractions of *P. wallachiana* against Foc

Treatment	Mean	Homogeneous Groups
8	24.400	A
4	23.800	A
2	21.000	B
6	18.600	C
1	0.0000	D
3	0.0000	D
5	0.0000	D
7	0.0000	D

Alpha 0.05 Standard Error for Comparison 0.8000
Critical T Value 2.037 Critical Value for Comparison 1.6295
There are 4 groups (A, B, etc.) in which the means
are not significantly different from one another.

Supplementary Table S5: Completely randomized analysis of variance for 1st severity scoring of banana plants drenched with different fractions of *P. wallachiana* in green house experiment. ($p< 0.05$)

Source	DF	SS	MS	F	P
Treatment	14	73.962	5.28299	13.2	0.0000
Error	90	36.000	0.40000		
Total	104	109.962			

Grand Mean 3.6476 CV 17.34

Supplementary Table S6: Pairwise Comparisons Test of 1st Severity Score for *P. wallachiana* fractions treatments used in green house experiment

Treatment	Mean	Homogeneous Groups
15	5.0000	A
13	4.5714	AB
14	4.4286	AB
1	4.2857	BC
3	4.2857	BC
7	4.2857	BC
10	4.0000	BCD
4	3.7143	CDE
9	3.5714	DE
2	3.4286	DE
12	3.2857	EF
11	2.7143	FG
6	2.5714	G

5	2.2857	G
8	2.2857	G

Alpha 0.05 Standard Error for Comparison 0.3381
Critical T Value 1.987 Critical Value for Comparison 0.6716
There are 7 groups (A, B, etc.) in which the means
are not significantly different from one another.

Supplementary Table S7: Completely randomized analysis of variance for 2nd severity scoring of banana plants drenched with different fractions of *P. wallachiana* in green house experiment. ($p < 0.05$)

Source	DF	SS	MS	F	P
Treatment	14	98.990	7.07075	20.6	0.0000
Error	90	30.857	0.34286		
Total	104	129.848			

Grand Mean 4.1048 CV 14.26

Supplementary Table S8: Pairwise Comparisons Test of 2nd Severity Score for *P. wallachiana* fractions treatments used in green house experiment

Treatment	Mean	Homogeneous Groups
1	5.0000	A
3	5.0000	A
4	5.0000	A
7	5.0000	A
10	5.0000	A
13	5.0000	A
15	5.0000	A

14	4.5714	AB
2	4.0000	BC
9	3.7143	CD
12	3.2857	DE
11	3.0000	EF
8	2.8571	EF
6	2.5714	F
5	2.5714	F

Alpha 0.05 Standard Error for Comparison 0.3130
Critical T Value 1.987 Critical Value for Comparison 0.6218
There are 6 groups (A, B, etc.) in which the means
are not significantly different from one another.

Supplementary Table S9: Completely randomized analysis of variance for 3rd severity scoring of banana plants drenched with different fractions of *P. wallachiana* in green house experiment. ($p< 0.05$)

Source	DF	SS	MS	F	P
Treatment	14	62.190	4.44218	8.91	0.0000
Error	90	44.857	0.49841		
Total	104	107.048			

Grand Mean 4.2381 CV 16.66

Supplementary Table S10: Pairwise Comparisons Test of 3rd Severity Score for *P. wallachiana* fractions treatments used in green house experiment

Treatment	Mean	Homogeneous Groups
1	5.0000	A

3	5.0000	A
4	5.0000	A
7	5.0000	A
10	5.0000	A
13	5.0000	A
15	5.0000	A
14	4.2857	AB
2	3.8571	B
12	3.7143	BC
5	3.5714	BC
9	3.5714	BC
11	3.5714	BC
8	3.0000	C
6	3.0000	C

Alpha 0.05 Standard Error for Comparison 0.3774
Critical T Value 1.987 Critical Value for Comparison 0.7497
There are 3 groups (A, B, etc.) in which the means
are not significantly different from one another.

Supplementary Table S11: Correlation coefficient and linear regression equation of each phenolic and flavanoid standards, at 285nm and 370nm, respectively.

Polyphenolic Standards	Linear regression equation via area	Correlation coefficient (R^2)	Level of detection LOD	Level of quantification LOQ
**Gallic acid**	y = 3550.6x+13254	0.9997	4.748764	14.39019
**Catechin**	y = 755x+2181.7	0.9976	13.41135	40.64045
**Epicatechin**	y = 1024x-3500.9	0.9969	15.15035	45.91015

**Coumeric acid**	y = 9443.8x+18646	0.9992	7.548394	22.87392
**Trans-Ferulic acid**	y =11042x-69020	0.9987	9.810637	29.7292
**Rutin**	y = 4407.7x+21012	0.9994	6.476182	19.62479
**Myrecitin**	y = 4417.6x+45057	0.999	8.343395	25.28301
**Quercetin**	y = 13890x-47681	0.9998	3.710513	11.24398
**Kaempferol**	y = 16883x-52784	0.9995	6.37866	19.32927

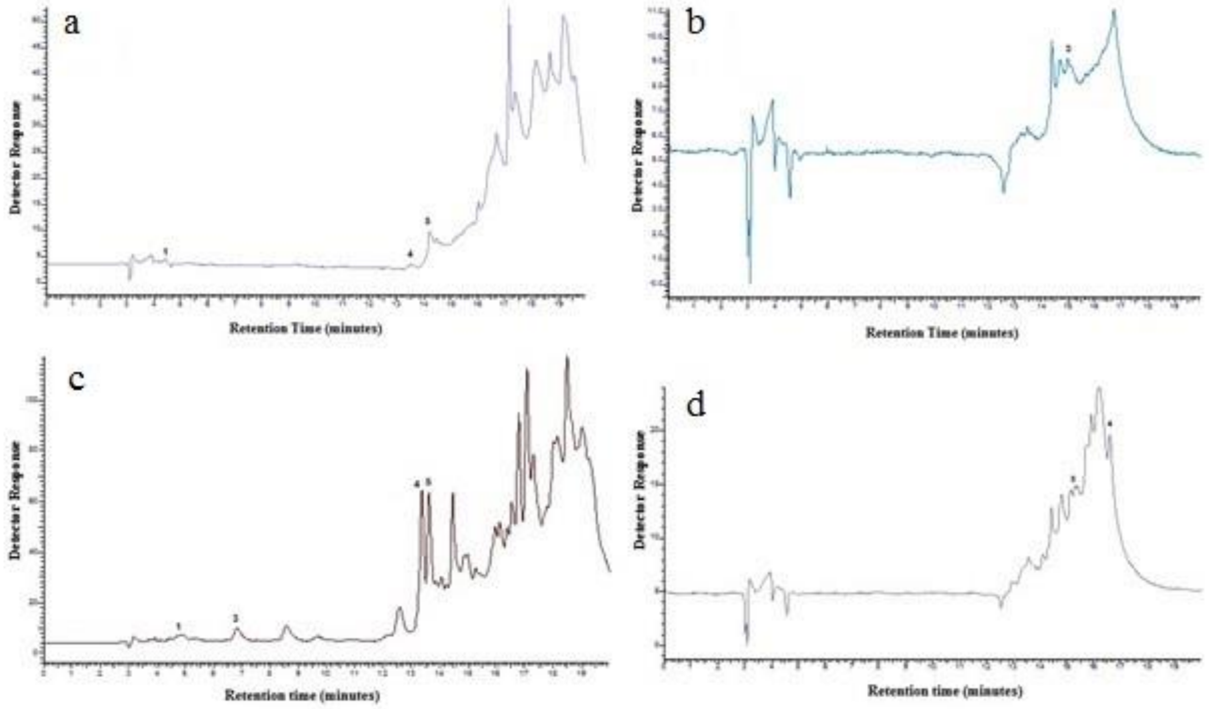

Supplementary Figure S1: HPLC chromatograms obtained for (a) n-hexane fraction at 285nm. 1=Gallic acid, 4=Coumeric acid, 5=trans-ferulic acid, (b) n-hexane fraction at 370 nm. 3=Quercitin, (c) Dichloromethane fraction at 285nm. 1= Gallic acid, 3=Epicatechin, 4=Coumeric acid, 5= trans-Ferulic acid, (d) Dichloromethane fraction at 370 nm. 3=Quercitin, 4= Kaempferol.
